# Effect of Xylitol and Maltitol Chewing Gums on Plaque Reduction and Salivary pH Modulation: A Retrospective Study in Pediatric Patients

**DOI:** 10.3390/dj13060233

**Published:** 2025-05-25

**Authors:** Francesco Saverio Ludovichetti, Edoardo Stellini, Camilla Rodella, Marco Sambugaro, Pier Francesco Gaja, Alessia Rizzato, Sergio Mazzoleni

**Affiliations:** Department of Neurosciences, Dentistry Section, Padova University, 35122 Padova, Italy; edoardo.stellini@unipd.it (E.S.); camilla.rodella@unipd.it (C.R.); marco.sambugaro@unipd.it (M.S.); pierfrancesco.gaja@studenti.unipd.it (P.F.G.); alessia.rizzato@studenti.unipd.it (A.R.); sergio.mazzoleni@unipd.it (S.M.)

**Keywords:** chewing gums, dental plaque, salivary pH, pediatric dentistry, xylitol, maltitol

## Abstract

**Objectives:** This clinical study aimed to evaluate the effects of two different sugar-free chewing gums on bacterial plaque reduction and salivary pH modulation in pediatric patients. **Methods:** This study retrospectively analyzed data from 100 children aged between 7 and 14 years who had previously participated in an investigation on plaque control and salivary pH. The Plaque Control Record Index (PCR) had been assessed in all participants using a plaque-disclosing agent, and salivary pH had been measured with a litmus test. The children had been randomly assigned into two groups: 50 had chewed a xylitol-containing gum, while the remaining 50 had chewed a maltitol-containing gum for five minutes. PCR and salivary pH values recorded immediately after chewing were reviewed for analysis. **Results:** Both chewing gums significantly reduced Plaque Control Record (PCR) values on dental surfaces (*p* < 0.001), indicating a statistically significant difference between pre- and post-treatment values. Salivary pH increased in both groups, suggesting a buffering effect associated with gum chewing. While the maltitol gum also reduced plaque accumulation, it showed greater variability in efficacy compared to the xylitol gum. No statistically significant differences were observed between the two gums in terms of salivary pH modulation. **Conclusions:** The findings highlight the beneficial effects of sugar-free chewing gums in reducing bacterial plaque and increasing salivary pH. These effects appear to be independent of the specific type of gum used, supporting their potential role as an adjunctive measure in oral hygiene maintenance.

## 1. Introduction

Dental caries is a chronic, infectious, and transmissible disease of multifactorial origin that affects the hard tissues of the teeth. The primary risk factors include bacterial plaque, diet, and host susceptibility, while additional elements such as salivary composition, oral hygiene habits, and general knowledge of oral health can influence the onset and progression of the disease [1].

The Global Burden of Disease Study has reported that untreated dental caries represents the most prevalent medical condition worldwide, with an estimated 520 million children affected in their primary dentition [2]. The World Health Organization (WHO) emphasizes that caries in six-year-old children is a major public health concern, with significant prevalence across different populations. In Italy, the prevalence of untreated dental caries in deciduous teeth among children aged 1–9 years is 36.1%, affecting approximately 1.8 million children [3].

Despite the multifactorial etiology of dental caries, the presence and dominance of cariogenic bacterial species in the oral biofilm remain a fundamental prerequisite for the development of the disease [4]. The oral biofilm is a highly structured microbial community composed of bacteria embedded in an extracellular polymeric matrix, firmly adherent to mucosal and dental surfaces [5]. Its formation begins immediately after toothbrushing, initially through weak physicochemical interactions, which subsequently evolve into stronger bonds that promote bacterial aggregation [6]. The early colonizers include *Streptococcus mitis* and *Streptococcus oralis*, while *Fusobacterium nucleatum* serves as a key bridge for co-adhesion within the biofilm structure [7].

Several environmental factors modulate the microbial composition and metabolism of the biofilm. The oral cavity maintains an average temperature of 35–37 °C, with slightly higher values in subgingival areas, which can favor the proliferation of anaerobic and periodontal pathogens [8]. Oral pH is another crucial determinant of bacterial activity. The buffering capacity of saliva, primarily mediated by bicarbonate and phosphate ions, plays a key role in maintaining a neutral pH. However, frequent acidification—such as that caused by the bacterial metabolism of dietary carbohydrates—favors the overgrowth of acidogenic and aciduric species like *Streptococcus mutans*, thereby promoting the carious process [9].

The initial phase of dental caries development occurs when *S. mutans* ferments dietary sugars, producing lactic acid and causing the pH to drop below the critical threshold of 5.5. This acidification leads to enamel demineralization, which clinically manifests as white spot lesions. Without adequate remineralization, these lesions progress into cavitated carious defects [10].

Saliva plays a fundamental role in modulating caries risk due to its ability to dilute sugars, neutralize acids, and facilitate enamel remineralization [11]. It consists of 99% water and 1% inorganic and organic components, including proteins, enzymes, and electrolytes, which contribute to its buffering properties. The bicarbonate–phosphate system is particularly crucial in maintaining oral pH, binding excess hydrogen ions to counteract acidity and restoring salivary pH to around 7.8 following an acid challenge [7].

Given the etiological role of bacterial plaque in caries development, its mechanical removal through oral hygiene practices is essential. Plaque disclosing agents, available in liquids to rinse or to apply on teeth by brush, gel, or tablet, can significantly enhance oral hygiene by making bacterial biofilm visible, thus improving plaque control efforts. Depending on their properties, they are classified as monochromatic, bitonal, tritonal, or fluorescent [8].

Alongside standard oral hygiene methods such as brushing, flossing, rinsing, and dietary management, chewing gum is a widely used adjunctive measure. Although William Semple, a dentist, patented a chewing gum formula in 1869, chewing gum has been used in various natural forms since ancient civilizations; it is primarily composed of three key ingredients: resin, wax, and elastomers, to which additional components are added to modify its physical and chemical properties [9]. According to the WHO, chewing gum provides multiple oral health benefits, including the mechanical removal of food debris and bacterial plaque and the stimulation of salivary flow, which enhances the buffering capacity and increases oral pH [10]. Some clinical studies suggest that this process may help rebalance the dynamic between enamel demineralization and remineralization, particularly in the early stages of caries development.

Most commercially available sugar-free chewing gums are sweetened with polyols, which have been classified as tooth-friendly by the European Community since 1994 [10]. The most commonly used polyols include erythritol, sorbitol, maltitol, and xylitol. Among these, xylitol has been shown to exert a significant anti-cariogenic effect by reducing the concentration of *Streptococcus mutans* and consequently decreasing the levels of lactic acid production [10]. Additionally, some studies suggest that maltitol may share certain properties with xylitol, particularly in its ability to reduce plaque accumulation and modulate the oral microbiota [11].

Many sugar-free chewing gums also contain fluoride, which has long been recognized for its role in caries prevention. An example of a sugar-free chewing gum containing fluoride is *Daygum Protex* (Perfetti Van Melle, Italy), formulated with fluoride, calcium, and xylitol to support enamel remineralization and caries prevention. Fluoride contributes to caries control by promoting the transformation of hydroxyapatite into fluorapatite, a more acid-resistant mineral phase, while also inhibiting bacterial colonization [10].

Despite these benefits, excessive gum consumption should be approached with caution, as it may lead to adverse effects such as excessive occlusal pressure on teeth, temporomandibular joint dysfunction, or the development of parafunctional habits. Furthermore, the presence of artificial sweeteners in some formulations may be associated with metabolic alterations, highlighting the importance of moderate consumption [10,11].

This retrospective study evaluates the effects of chewing gum on bacterial plaque reduction and salivary pH modulation in a pediatric population. Specifically, we analyze previously collected data to compare the efficacy of xylitol-containing and maltitol-containing gums in influencing these parameters. The null hypothesis of this study was that there would be no significant differences in plaque reduction or salivary pH modulation between xylitol-containing and maltitol-containing chewing gums.

## 2. Materials and Methods

This retrospective study analyzed previously collected data from a cohort of 100 children aged between 7 and 14 years who had independently chewed sugar-free gum. The aim was to assess the effects of xylitol- and maltitol-containing gums on bacterial plaque reduction and salivary pH modulation. This study was conducted in accordance with the Declaration of Helsinki, and written informed consent was obtained from all parents or legal guardians prior to data collection.

The data were obtained from records of children who met specific inclusion criteria: good general health, no systemic diseases affecting salivary composition (e.g., diabetes, Sjögren’s syndrome), and no recent use of antibiotics or professional fluoride applications in the past three months. Exclusion criteria included the presence of orthodontic appliances, active periodontal disease, or known allergies to any component of the chewing gums. Children were recruited from private dental clinics in Northern Italy as part of routine check-ups, and only those who met all inclusion criteria were considered. The children were randomly assigned to the xylitol or maltitol group using a simple randomization list generated by the research team.

Participants were divided into two groups based on the type of gum they had reported using:

- Xylitol gum group (*n* = 50): Children who had chewed a sugar-free gum containing xylitol as the primary sweetener.
- Maltitol gum group (*n* = 50): Children who had chewed a sugar-free gum containing maltitol as the primary sweetener.

The xylitol chewing gum used was Vivident Xylit, manufactured by Perfetti Van Melle, Lainate, Italy. The maltitol chewing gum used was Daygum Protex, also manufactured by Perfetti Van Melle, Lainate, Italy.

The mean age of the participants was 10.3 years (range: 7–14 years), with a gender distribution of 52 males and 48 females.

The mean age was comparable between groups: 10.4 ± 2.1 years in the xylitol group and 10.2 ± 2.3 years in the maltitol group. Gender distribution was also similar: 26 males and 24 females in the xylitol group, 26 males and 24 females in the maltitol group.

The xylitol gum was marketed as an anti-plaque chewing gum, while the maltitol gum did not have specific anti-plaque claims.

Bacterial plaque levels were assessed using the Plaque Control Record Index (PCR), Mira-2-ton^®^ (Hager & Werken GmbH & Co. KG, Duisburg, Germany) was used as the plaque-disclosing agent, and it was applied to highlight areas with bacterial biofilm. All PCR evaluations were performed by two calibrated researchers (inter-examiner kappa = 0.89), trained prior to this study to ensure scoring consistency. Each participant rinsed with the disclosing agent, and plaque presence was recorded as a percentage of stained surfaces per total examined surfaces.

Salivary pH was measured using pH test strips (Macherey-Nagel GmbH & Co. KG, Düren, Germany; sensitivity range 5.0–8.0) to determine the baseline acidity of the oral cavity. Saliva samples were collected following a standardized protocol:

1. Participants refrained from eating or drinking for at least 30 min before sample collection.
2. Unstimulated saliva was collected in a sterile container by having the child passively drool for 30 s.
3. A pH strip was immersed in the saliva sample for 10 s, and the pH value was recorded.

Participants chewed the assigned gum continuously for approximately five minutes, under supervision, as per the standardized protocol. All chewing sessions were directly supervised by trained members of the research team to ensure correct execution and compliance. Children were previously instructed on how to chew the gum correctly and were observed during the chewing period by a researcher to ensure compliance. A post-chewing salivary pH measurement was recorded using the same pH test strips to observe any changes in salivary pH. Similarly, a second Plaque Control Record Index (PCR Index) assessment was conducted with the same plaque disclosing agent to evaluate any reduction in bacterial biofilm accumulation.

Data were analyzed using SPSS Statistics for Windows, Version 27.0 (IBM Corp., Armonk, NY, USA). Normality of the data distribution was assessed using the Shapiro–Wilk test.

- Intragroup comparisons (pre- and post-intervention for each group) were performed using the Wilcoxon signed-rank test, given the non-parametric nature of the data.
- Intergroup comparisons (between xylitol and maltitol groups) were conducted using the Mann–Whitney U test.
- A *p*-value < 0.05 was considered statistically significant.

## 3. Results

### 3.1. PCR Index

#### 3.1.1. Xylitol Group

**Figure 1:**
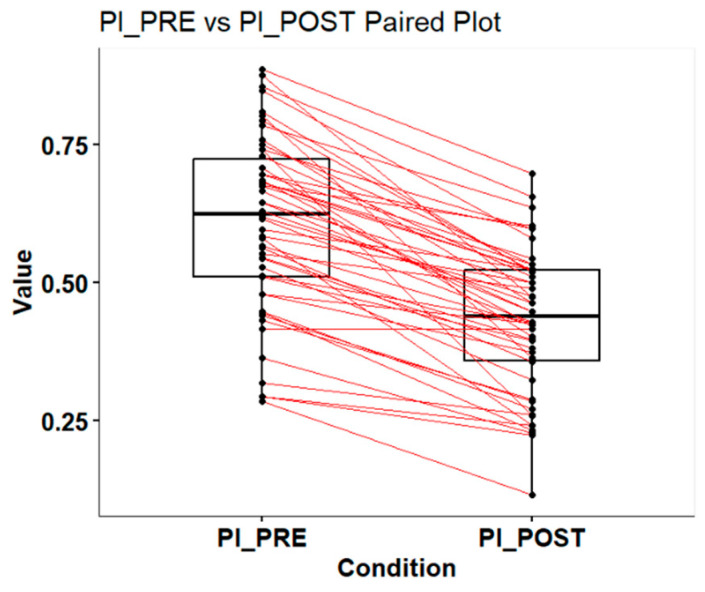
Reduction in PCR Index before and after chewing xylitol gum. PI_PRE = PCR Index before chewing; PI_POST = PCR Index after chewing.

The included boxplots (Figure 1) display the distribution of the data for (PCR Index pre-chewing) PI_PRE and (PCR Index post-chewing) PI_POST. The thick horizontal line in each box represents the median of the data. The box itself represents the interquartile range (IQR), which is the range between the first quartile (25th percentile) and the third quartile (75th percentile). The lines extending from the boxes (whiskers) represent the data range, typically 1.5 times the IQR. Any point outside the whiskers is considered an outlier and is displayed as an individual point.

Most of the lines slope downward, indicating that the majority of subjects or groups experienced a reduction in the PCR Index after intervention. The PI_PRE data show greater variation than PI_POST, suggesting higher variability in the initial PCR Index values. After intervention, the values appear more concentrated around a lower PCR Index. The median PCR Index is lower in the PI_POST group compared to the PI_PRE group, indicating that it was effective in reducing the PCR Index for most subjects or groups. Therefore, the graph suggests that the intervention was generally effective in reducing the PCR Index from PI_PRE to PI_POST. The downward slope of the lines and the decrease in both the median and the dispersion of PI_POST values support this conclusion.

In Table 1, some descriptive measures can be verified by comparing pre- and post-intervention values. The minimum values indicate that the lowest PCR Index before intervention was 0.2857, whereas, after intervention, it reduced to 0.1161. The first quartile (1st Qu.) represents the value below which 25% of the data points fall, showing a reduction from 0.5108 to 0.3589 from pre- to post-intervention. The median, which is the central value of the data, also significantly decreased from 0.6243 to 0.4382, indicating an overall improvement in the PCR after intervention.

The average PCR before intervention was 0.6095, whereas, after intervention, this average dropped to 0.4312, reinforcing the trend of a reduction in the PCR. The third quartile (3rd Qu.), which represents the value below which 75% of the data points fall, shows a decrease from 0.724 to 0.5224. Finally, the maximum values of the PCR also decreased, from 0.8854 in the pre-treatment phase to 0.6979 in the post-intervention phase. In summary, all the statistical parameters presented indicate a reduction in the PCR after intervention, suggesting that the intervention was effective in improving the plaque condition of the subjects or groups analyzed.

In this study, we aimed to assess the effectiveness of a new product for preventing dental plaque buildup. To do so, we compared plaque levels before and after the use of the product “ANTI-PLAQUE GUM WITH XYLITOL” in a group of patients. We used the Wilcoxon signed-rank test, a statistical method that compares two measurements taken from the same group of individuals at different times. This test is particularly useful for determining whether there is a significant difference between the two sets of data, given that the data are paired and may not follow a normal distribution. The analysis was conducted with data collected before (PI_PRE) and after (PI_POST) using the product, ensuring an accurate and direct comparison.

The analysis revealed a *p*-value of less than <0.001, indicating a highly significant difference between plaque levels before and after using the product. In other words, the results suggest that the product has a positive effect on reducing dental plaque, with a statistically significant difference.

#### 3.1.2. Maltitol Group

**Figure 2:**
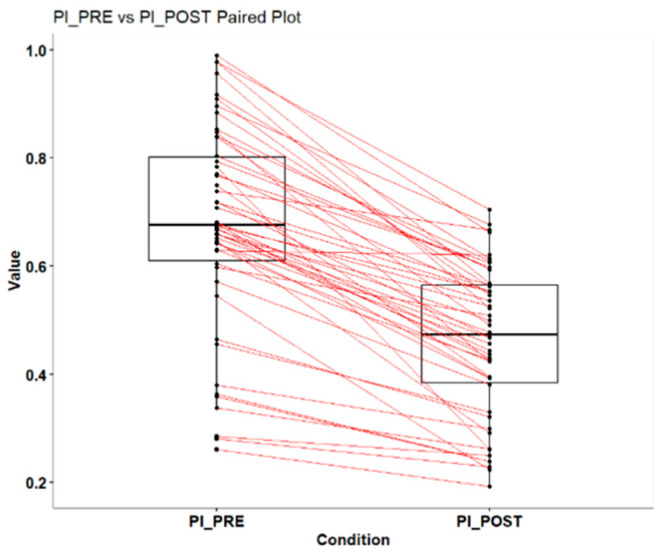
Reduction in PCR Index before and after chewing maltitol gum.

As with the previous material, a significant reduction in the PCR Index was observed. The analysis of the dental plaque data, both before and after intervention, shows a clear reduction in plaque levels. Before intervention (PI_PRE), the plaque percentage ranged from 19% to 99%, with a mean of 47% and a median of 46%. After intervention (PI_POST), the percentage range was between 26 and 70%, with a mean of 47% and a median of 46% (Table 2). While the median remained constant, the range of values and the first quartile showed a reduction in the plaque level after intervention. These data indicate that the intervention had a positive effect in reducing the amount of plaque, improving plaque control in most patients (Figure 2).

To determine the impact of the intervention on the reduction in dental plaque, we applied the Wilcoxon test for paired samples with continuity correction. The results revealed a V value of 1275 and a *p*-value lower than <0.001, indicating a statistically significant difference in plaque levels before and after the intervention. This extremely low *p*-value suggests that the observed reduction in plaque did not occur by chance, confirming the effectiveness of the intervention in significantly reducing the amount of dental plaque. Therefore, the results strengthen the conclusion that the intervention was effective in improving plaque control for the majority of the patients analyzed.

### 3.2. pH

#### 3.2.1. Xylitol Group

**Figure 3:**
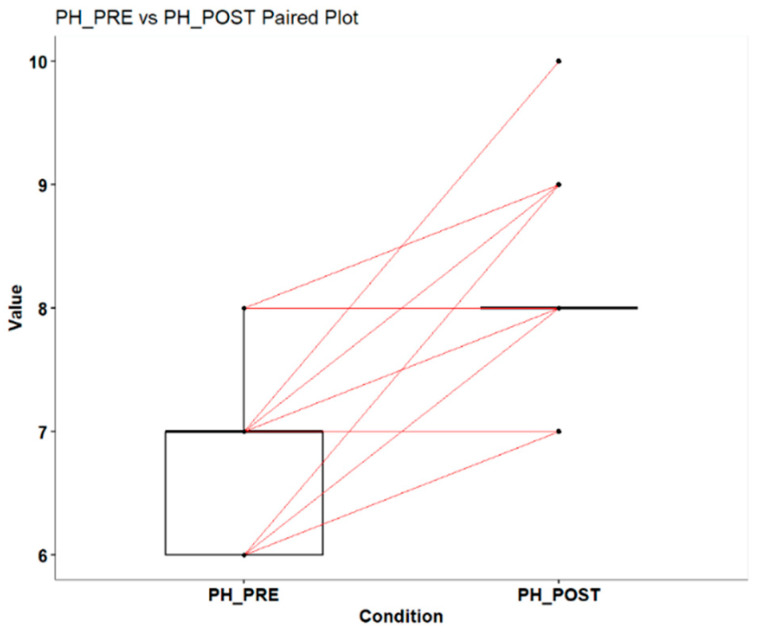
Changes in salivary pH before and after chewing xylitol gum.

As shown in Figure 3 and Table 3, before the intervention, the pH ranged from 6 to 8, with the median at 7 and the mean at 6.86. After the intervention, a consistent increase was observed, with the pH now ranging from 7 to 10. The median and mean increased to 8, indicating an overall increase in pH. The first quartile and median values after intervention are higher than the values before intervention, suggesting that most patients experienced an increase in pH. This shift to a less acidic oral environment may indicate an improvement in oral health, potentially reducing the incidence of dental problems.

To assess the effectiveness of the intervention in altering oral pH levels, we used the Wilcoxon signed-rank test for paired data. The results of this test revealed a *p*-value of <0.001, indicating a highly significant difference between the pH levels before and after the intervention. This suggests that the observed change in pH is not due to chance and that the intervention had a substantial effect on increasing oral pH. In summary, the test confirms that there was an increase in pH value, and this difference is statistically significant.

#### 3.2.2. Maltitol Group

**Figure 4:**
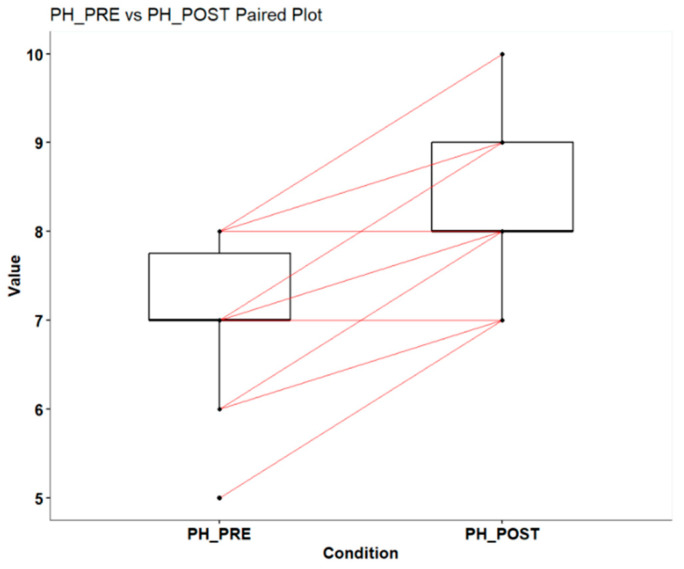
Changes in salivary pH before and after chewing maltitol gum.

The analysis of pH levels in the NON ANTI-PLAQUE WITH MALTITOL group, before and after intervention, indicates a significant improvement in the patients’ oral acidity. Before intervention (PH_PRE), pH values ranged from 5 to 8, with a median and average of 7, reflecting a neutral environment. The first quartile was at 7, while the third quartile reached 8, showing a moderate distribution of pH in the lower range. After intervention (PH_POST), pH levels increased, with values ranging from 7 to 10. The median and average after intervention rose to 8, demonstrating a consistent shift towards an alkaline environment. The first quartile increased to 8, and the third quartile reached 9, showing an overall increase in pH levels (Figure 4) (Table 4).

Finally, the Wilcoxon paired sample test produced a V value of 0 and a *p*-value of 1.608 × 10^−8^, indicating a highly significant difference between pH levels before and after intervention. This extremely low *p*-value suggests that the change in pH is not due to chance and confirms that the intervention was effective in raising pH levels. In summary, the data show that the intervention significantly improved the oral acid–base balance.

### 3.3. Plaque Assesment

At baseline, mean PCR values were 60.9% (xylitol) and 68% (maltitol), while mean salivary pH values were 6.86 and 7.0, respectively. These differences were not statistically significant (*p* > 0.05)

#### 3.3.1. PCR Index

**Figure 5:**
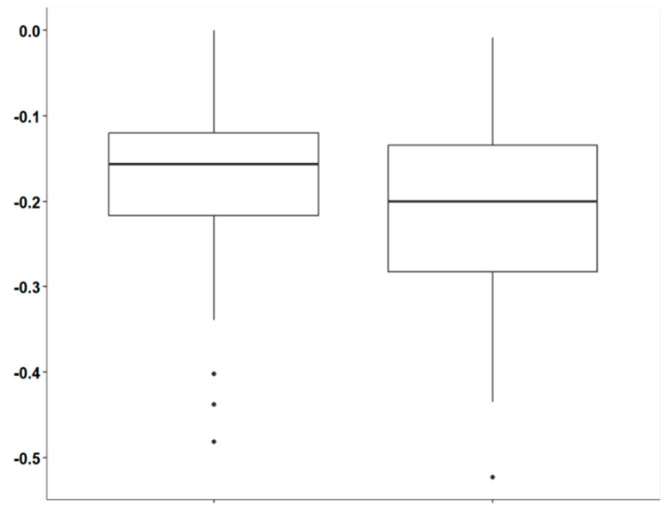
Comparison of PCR Index reduction between xylitol (**left**) and maltitol (**right**) gums.

Now performing an analysis between the groups, for the comparative analysis between the interventions “ANTI-PLAQUE GUM WITH XYLITOL” and “NON-ANTI-PLAQUE GUM WITH MALTITOL”, the difference in PCR Index (diff_PI) for each group was calculated. The difference (diff_PI) was obtained by subtracting the post-intervention PI value from the pre-intervention PI value (Figure 5). This calculation was essential to compare the effectiveness of the interventions, as it directly allows for the evaluation of each treatment’s impact on the reduction in the PI index. As a result, the diff_PI data for each group are independent and represent the specific variation in each intervention, facilitating a clear and objective comparison between the two interventions. This approach ensures that the analysis of the differences between the groups only considers the intervention effect.

We can therefore observe that, for the treatments “ANTI-PLAQUE GUM WITH XYLITOL” and “NON-ANTI-PLAQUE GUM WITH MALTITOL”, the descriptive statistics reveal significant differences in the reduction in the PI index (diff_PI). The intervention with “ANTI-PLAQUE GUM WITH XYLITOL” shows an average reduction of −0.178, with a relatively low variation (standard deviation of 0.0992), and diff_PI values ranging from −0.481 to 0. The group has a median of −0.157 and a negative skew of −0.987, suggesting that most of the data are concentrated to the right of the mean and that there is a consistent reduction, although extreme values may exhibit slight variations. In contrast, the “NON-ANTI-PLAQUE GUM WITH MALTITOL” group shows a slightly greater average reduction of −0.214, with greater variation (standard deviation of 0.113) and values ranging from −0.523 to −0.0084. The median is −0.201, with a less pronounced skew of −0.497, indicating a more symmetrical distribution compared to the previous group (Table 5). The greater variance in this group suggests that the values are more dispersed around the mean, reflecting a more varied response to the intervention. These differences indicate that, although both treatments lead to a reduction in the PI index, “NON-ANTI-PLAQUE GUM WITH MALTITOL” tends to have a more pronounced average reduction, but with greater variability in the results.

To verify if there is a statistically significant difference in the reduction between the two groups, we applied the Wilcoxon rank-sum test (or Mann–Whitney test) for unpaired data. This test was performed to compare the differences in the PI index (diff_PI) between the two treatment groups, “ANTI-PLAQUE GUM WITH XYLITOL” and “NON-ANTI-PLAQUE GUM WITH MALTITOL”. The alternative hypothesis is that there is a significant difference. With a *p*-value of 0.08542 and using a significance level of 10%, there is sufficient evidence to reject the null hypothesis. This suggests that there is a statistically significant difference between the interventions “ANTI-PLAQUE GUM WITH XYLITOL” and “NON-ANTI-PLAQUE GUM WITH MALTITOL” in their impact on the PI index (diff_PI). In other words, the analysis indicates that the two treatments have different effects on the PI index, although the difference is not as strong as would be suggested by a 5% significance level. Therefore, with a more flexible significance level, the observed difference between the groups is considered statistically significant.

#### 3.3.2. pH

The analysis of the pH differences (diff_PH) between the treatments “ANTI-PLAQUE GUM WITH XYLITOL” and “NON-ANTI-PLAQUE GUM WITH MALTITOL” highlights some significant differences. For “ANTI-PLAQUE GUM WITH XYLITOL”, the average pH difference is 1.14, with a standard deviation of 0.808, indicating an average increase in pH with considerable variation between the data. The minimum observed value was 0, while the maximum was 3, suggesting that, in some cases, there was no change in pH, while, in others, a significant increase occurred. The median is 1, with the 25th and 75th percentiles (q25 and q75) at 1 and 2, respectively, indicating that most of the data are concentrated between these values. The skewness of 0.205 suggests a slight rightward tilt, with a variance of 0.653 confirming the observed dispersion.

For “NON-ANTI-PLAQUE GUM WITH MALTITOL”, the average pH difference is slightly higher at 1.28, with a standard deviation of 0.809, similar to the previous group, indicating comparable variation in the data. The minimum value is also 0, while the maximum is 2, indicating a lower maximum increase compared to the “ANTI-PLAQUE GUM WITH XYLITOL” group. The median is 1.5, with the 25th and 75th percentiles (q25 and q75) at 1 and 2, respectively. The skewness of −0.53 suggests a slight leftward tilt, indicating that most values are concentrated on the right side of the distribution. The variance of 0.655 is similar to that of the other group, confirming the observed dispersion in the data.

**Figure 6:**
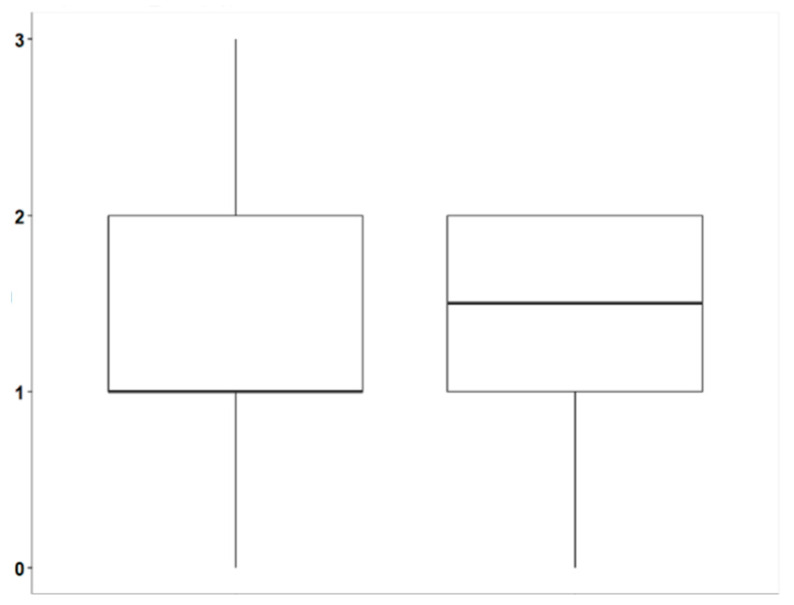
Comparison of salivary pH changes between xylitol (**left**) and maltitol (**right**) gums.

Considering these statistics, both treatments lead to pH increases, but “NON-ANTI-PLAQUE GUM WITH MALTITOL” tends to show a slightly higher average, although with a more concentrated and symmetric distribution compared to “ANTI-PLAQUE GUM WITH XYLITOL” (Figure 6) (Table 6).

To compare the pH differences (diff_PH) between the treatment groups “ANTI-PLAQUE GUM WITH XYLITOL” and “NON-ANTI-PLAQUE GUM WITH MALTITOL”, the Wilcoxon rank-sum test (or Mann–Whitney test) was performed. The *p*-value of 0.2702 is greater than the commonly used significance levels of 0.05 and 0.10. This indicates that there is insufficient statistical evidence to reject the null hypothesis, which states that there is no significant difference in the pH differences (diff_PH) between the two treatment groups. In summary, based on the results of the Wilcoxon test, we did not find a statistically significant difference between the treatments “ANTI-PLAQUE GUM WITH XYLITOL” and “NON-ANTI-PLAQUE GUM WITH MALTITOL” in their impact on pH. Therefore, the observed variations in pH after the interventions cannot be considered statistically significant differences between the two groups.

## 4. Discussion

The present clinical study aimed to assess whether chewing gums could effectively reduce bacterial plaque on dental surfaces and increase salivary pH, serving as a potential adjunct to standard at-home oral hygiene practices. Furthermore, this study sought to evaluate whether there were significant differences in efficacy between a commercially available xylitol-containing gum labeled as “anti-plaque” and a maltitol-containing gum not specifically marketed for plaque control. Chewing gum may be considered a complementary tool for caries prevention, but it should not replace conventional preventive measures such as tooth brushing.

Several studies support the effectiveness of sugar-free chewing gum in reducing plaque accumulation [12]. While chewing gum alone does not provide a substitute for proper oral hygiene, its adjunctive use, particularly when combined with regular toothbrushing, has been associated with a reduction in residual plaque, especially on occlusal surfaces [13]. A review published in the *Journal of the Irish Dental Association* in 2012 highlighted the oral health benefits of chewing gum, emphasizing its role in stimulating salivary flow, neutralizing plaque acidity through its buffering capacity, and enhancing the remineralization of early carious lesions [14]. Additionally, epidemiological data indicate a lower incidence of dental caries in children who routinely chew sugar-free gum [15].

Chewing gum can thus be considered a complementary tool for dental disease prevention, but it should not replace conventional preventive measures. One of its notable advantages is the ability to modulate the oral microbiota by reducing cariogenic bacterial loads. In particular, several studies indicate that chewing gum contributes to a significant decrease in *Streptococcus mutans* levels in the oral cavity. A systematic review and meta-analysis conducted in 2021 examined variations in S. mutans concentrations among adults and children who chewed sugar-free gum, placebo gum, or other products such as probiotics. The findings confirmed that sugar-free gum significantly reduced S. mutans levels compared to non-chewing controls [15].

In the present study, both xylitol and maltitol chewing gums resulted in a statistically significant reduction in bacterial plaque among participants, with most showing a lower PCR Index after the intervention. Similarly, both groups exhibited a statistically significant increase in salivary pH, indicating a shift toward a neutral or alkaline oral environment and, consequently, a reduced risk of enamel demineralization. These findings are consistent with a randomized controlled study conducted in 2011, in which fluoride-containing and placebo chewing gums were administered for four weeks to subjects wearing removable orthodontic appliances with demineralized human enamel specimens. The study found that fluoride-containing gum promoted greater remineralization and acid resistance than the placebo [16].

Regarding the comparison between the two test groups, both xylitol- and maltitol-containing gums significantly reduced plaque accumulation. However, although the maltitol gum group exhibited a greater variability in outcomes, its effectiveness was comparable to that of the xylitol gum. Moreover, no statistically significant differences were observed between the two groups concerning salivary pH modulation.

The literature presents conflicting evidence regarding the superiority of xylitol over other polyols. While some studies suggest that xylitol has superior cariostatic effects, others report no significant difference between xylitol and maltitol. A comparative study from 2000 investigated the effects of chewing gums containing xylitol, sorbitol, sucrose, or maltitol over 14 days in 13 adults. While significant differences were found between xylitol and sorbitol or sucrose in terms of plaque accumulation and acidity, no differences were observed between xylitol and maltitol [17].

Similarly, a 2013 study demonstrated that both xylitol- and maltitol-containing gums could reduce plaque, decrease oral acidity, and lower the concentrations of four major cariogenic bacterial species (*S. mutans*, *S. sobrinus*, *A. viscosus*, and *Lactobacillus*) in a comparable manner [18].

Nonetheless, other studies favor xylitol over alternative sweeteners. A 2015 study on 32 adults with poor oral hygiene habits evaluated the effects of xylitol- and sucrose-containing gums on salivary *S. mutans* levels. The results showed that xylitol gum was significantly more effective in reducing bacterial load compared to the sucrose-containing gum [19]. Similarly, a randomized controlled trial conducted on 60 children assigned to chew xylitol or other polyol gums twice daily for 30 days found that the xylitol gum provided the greatest reduction in *S. mutans* levels, reinforcing its potential as a preventive strategy alongside standard oral hygiene practices [20].

Beyond its effects on *S. mutans*, xylitol may also exert a broader antimicrobial influence on periodontal pathogens. A 2022 study involving 24 adults compared individuals who chewed xylitol gum with those who did not. After two weeks, participants in the xylitol gum group exhibited a 20% reduction in bacterial plaque, along with a decrease in Firmicutes (10.26%), a bacterial phylum linked to caries, and periodontal pathogens Bacteroidetes (6.32%) and Actinobacteria (1.66%) [21].

Despite promising evidence, some reviews suggest that additional research is required to substantiate the long-term anticariogenic effects of xylitol. A 2012 systematic review comparing xylitol to fluoride, the current gold standard in caries prevention, concluded that, while xylitol supplementation may enhance caries prevention, further clinical trials are necessary to strengthen the evidence and compare its effects with other sugar substitutes [22].

Notably, in the present study, the labeling of chewing gums as “anti-plaque” or “non-anti-plaque” did not appear to influence their effectiveness. Regardless of branding, sugar-free gums contribute to plaque removal primarily through mechanical action and contain various bioactive ingredients that may further enhance their benefits. Additionally, no gum was found to be superior in removing bacterial plaque, even those containing xylitol. This aligns with findings from a 2016 study that analyzed commercially available chewing gums in Asian markets. The study revealed that most gums do not provide a sufficient xylitol concentration to exert a significant cariostatic effect, reinforcing the notion that their benefits are largely attributable to their sugar-free composition rather than xylitol content alone [23].

This study has several limitations. First, its retrospective design may introduce selection bias, and randomization procedures rely on historical allocation. Second, the sample size was relatively small and limited to a single center, which may affect the generalizability of the results. Third, the short chewing duration (five minutes) reflects only immediate effects; longer-term effects were not assessed. Additionally, the PCR Index and salivary pH were measured using semi-quantitative tools, which may lack the precision of more advanced analytical methods. Future prospective studies with larger populations and longer follow-up are needed to confirm these findings.

## 5. Conclusions

This study confirms that sugar-free chewing gums can help reduce plaque accumulation and increase salivary pH in pediatric patients after just five minutes of use. Both xylitol and maltitol gums were effective, with maltitol showing a slightly greater average reduction in PCR and xylitol providing more consistent results. These findings suggest that sugar-free gums, regardless of composition, can be a useful adjunct to oral hygiene but should not replace tooth brushing. Short-term use appears beneficial, though further research is needed to evaluate long-term effects and optimize formulations.

## Figures and Tables

**Table 1:** Descriptive measures: PI_PRE = PCR Index before chewing; PI_POST = PCR Index after chewing.

	PI_PRE	PI_POST
Min	29%	12%
1st Qu	51%	36%
Median	62%	44%
Mean	61%	43%
3rd Qu	72%	52%
Max	89%	70%

**Table 2:** Descriptive analysis considering “NON ANTI-PLAQUE GUM WITH MALTITOL”.

	PI_PRE	PI_POST
Min	26%	19%
1st Qu	61%	38%
Median	68%	47%
Mean	68%	46%
3rd Qu	80%	56%
Max	99%	70%

**Table 3:** Descriptive analysis of pH.

	PI_PRE	PI_POST
Min	6	7
1st Qu	6	8
Median	7	8
Mean	7	8
3rd Qu	7	8
Max	8	10

**Table 4:** Descriptive measures: NON-ANTI-PLAQUE GUM WITH MALTITOL.

	PH_PRE	PH_POST
Min	5	7
1st Qu	7	8
Median	7	8
Mean	7	8
3rd Qu	8	9
Max	8	10

**Table 5:** Descriptive measures of PCR Index reduction between xylitol (left) and maltitol (right) gums.

	ANTI-PLAQUE GUM WITH XYLITOL	NON-ANTI-PLAQUE GUM WITH MALTITOL
Mean	−0.178	−0.214
Sd	0.0992	0.113
Min	−0.481	−0.523
Max	0	−0.0084
Median	0.157	−0.201
q25	−0.217	−0.283
q75	−0.12	−0.134
Skewness	−0.987	−0.497
Variance	0.00983	0.0128

**Table 6:** Descriptive measures of salivary pH changes between xylitol (left) and maltitol (right) gums.

	ANTI-PLAQUE GUM WITH XYLITOL	ANTI-PLAQUE GUM WITH MALTITOL
Mean	1.14	1.28
Sd	0.808	0.809
Min	0	0
Max	3	2
Median	1	1.5
q25	1	1
q75	2	2
Skewness	0.205	−0.53
Variance	0.653	0.655

## Data Availability

The raw data supporting the conclusions of this article will be made available by the authors on request.
